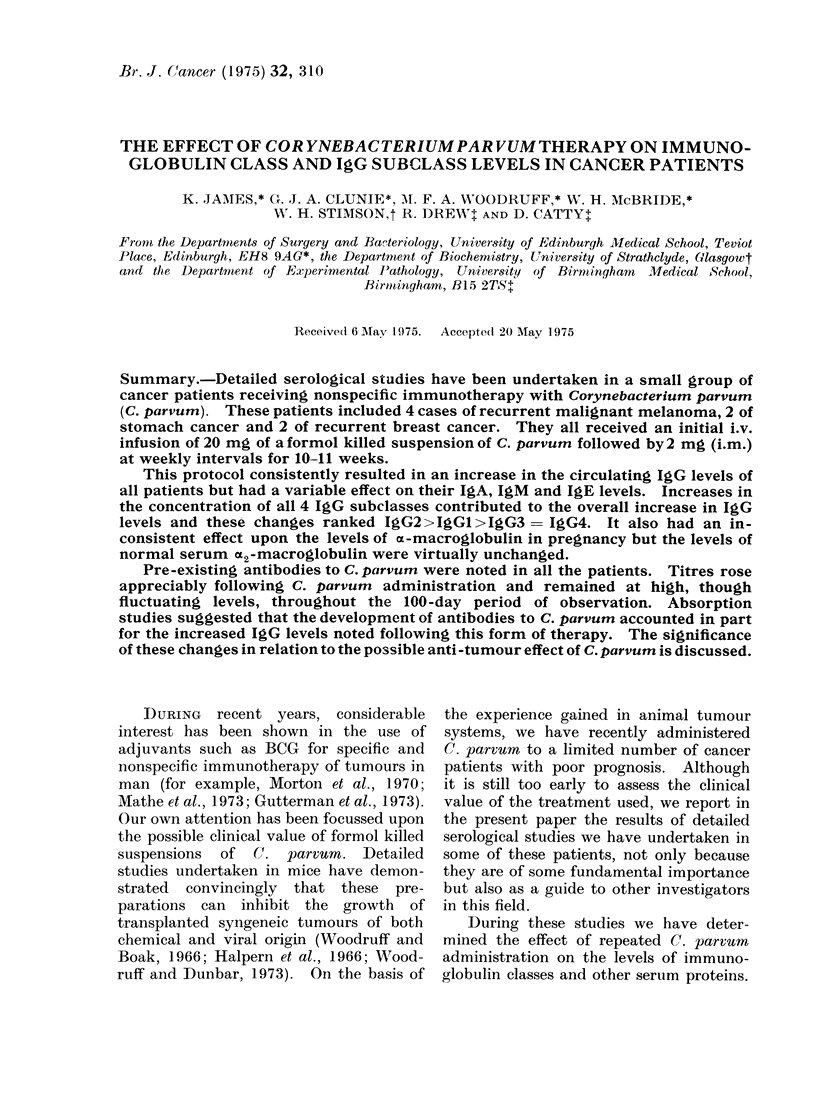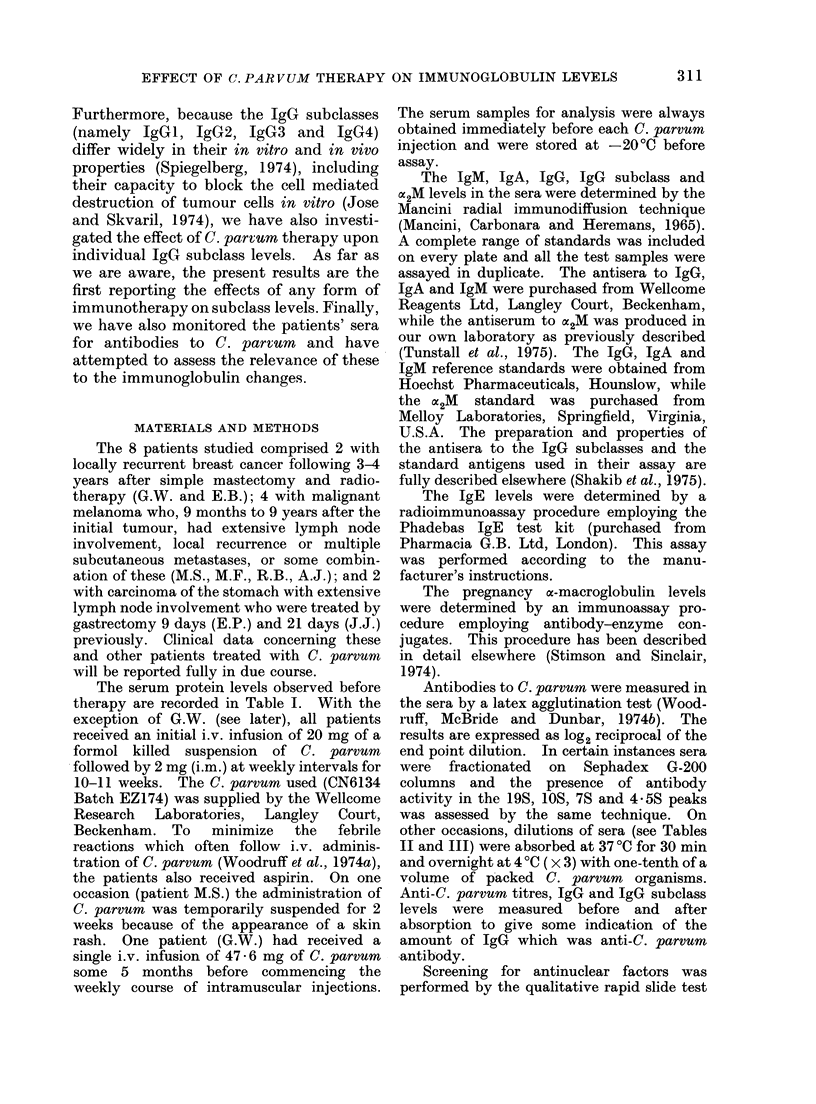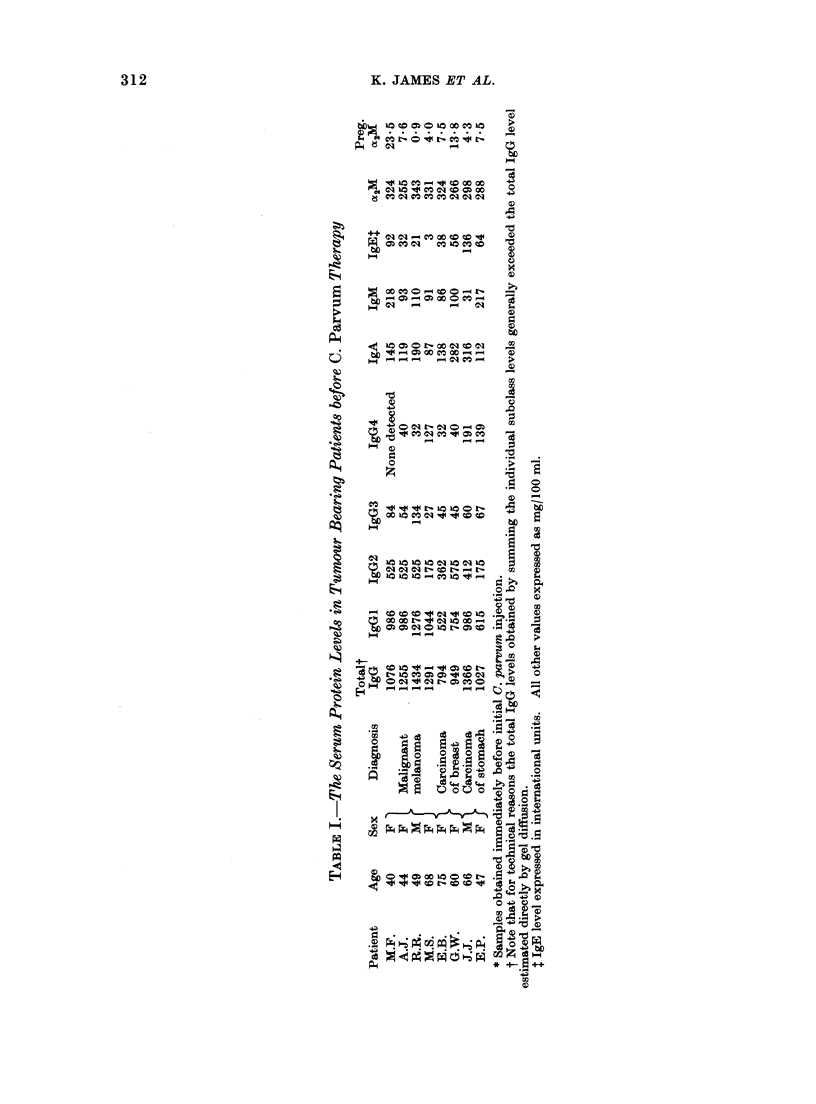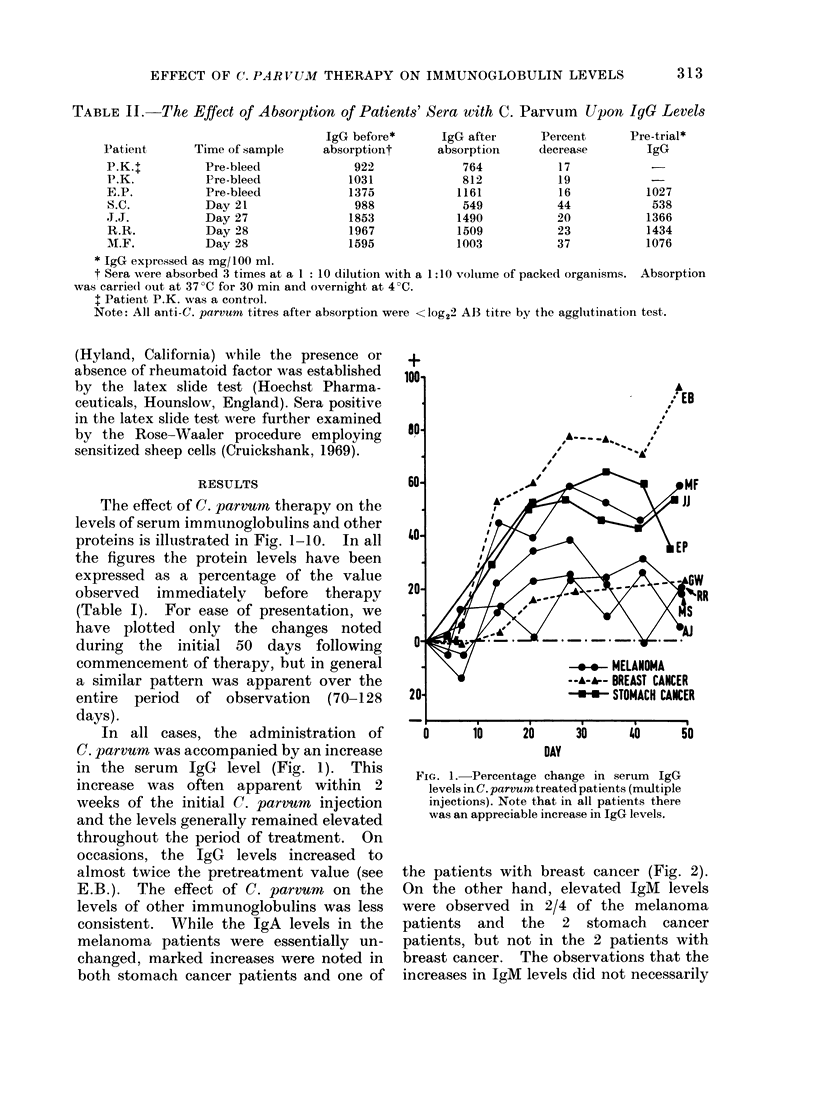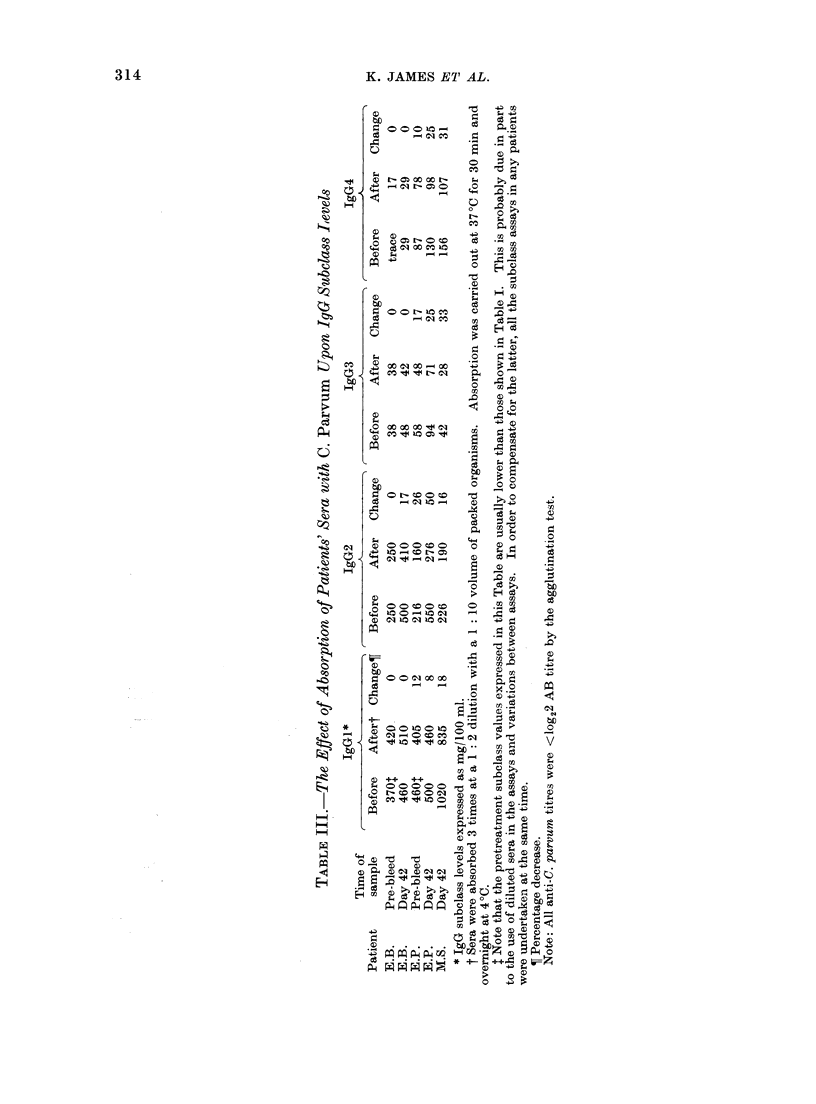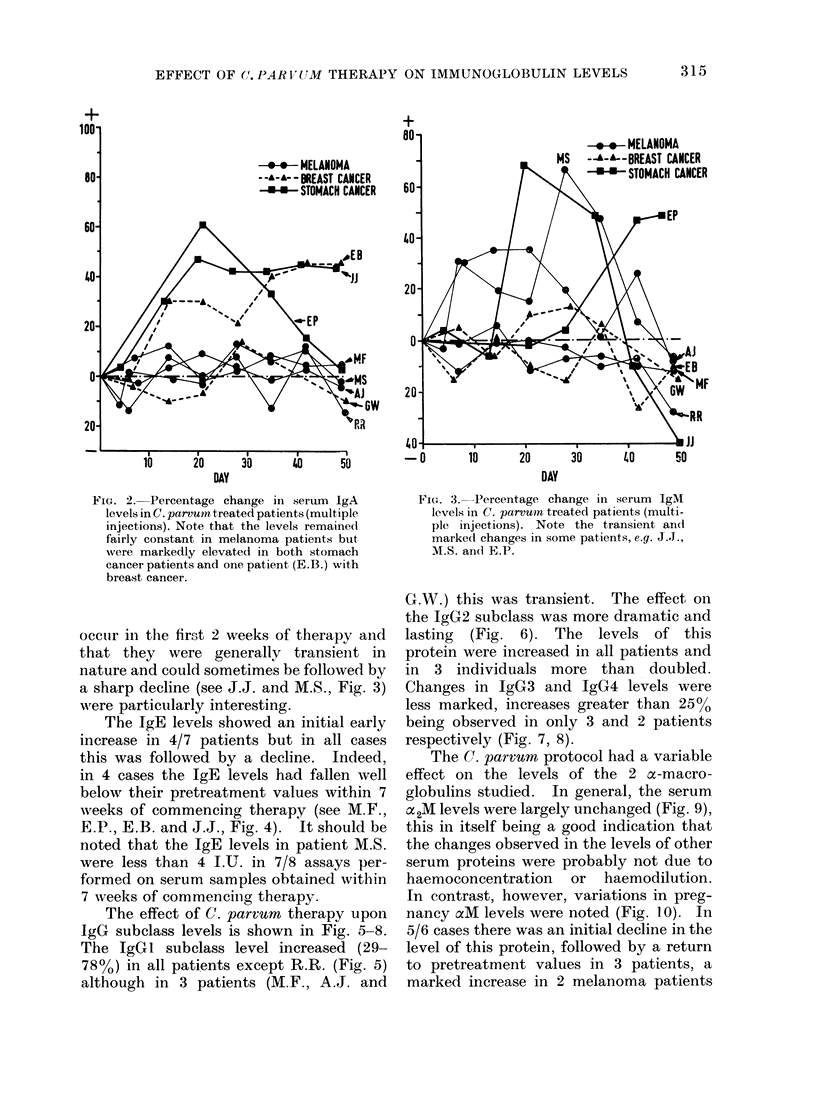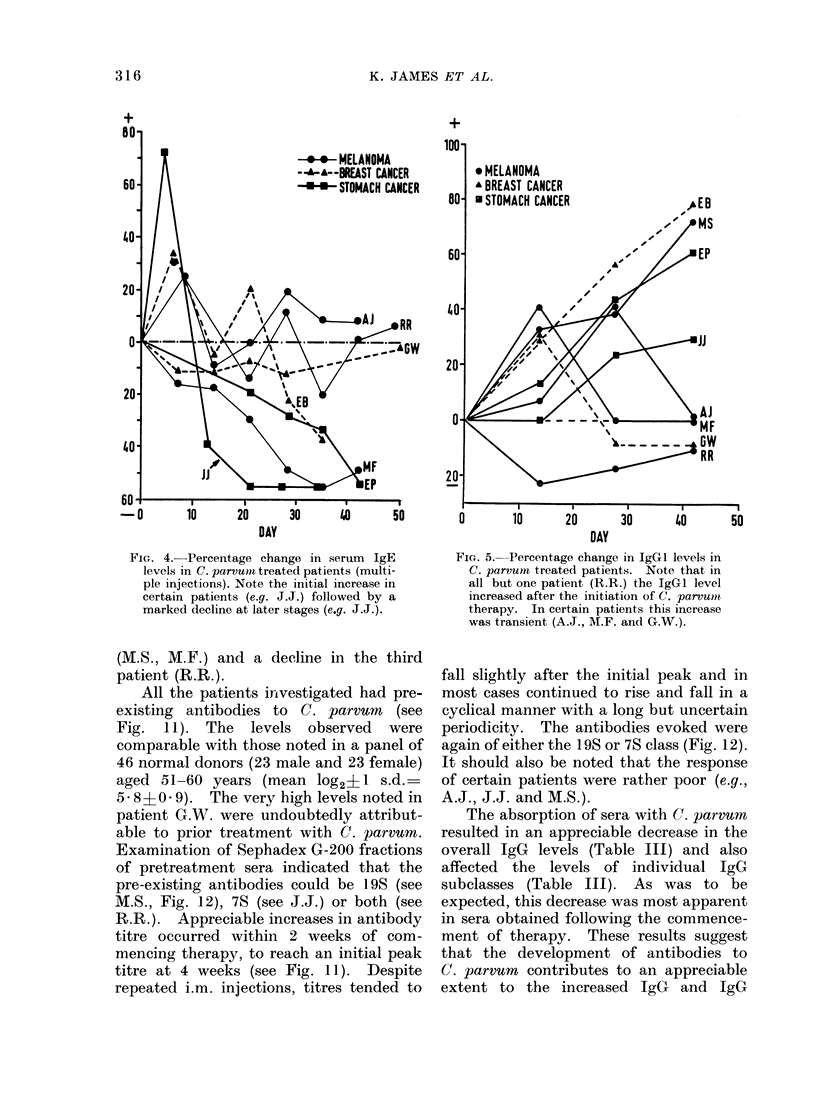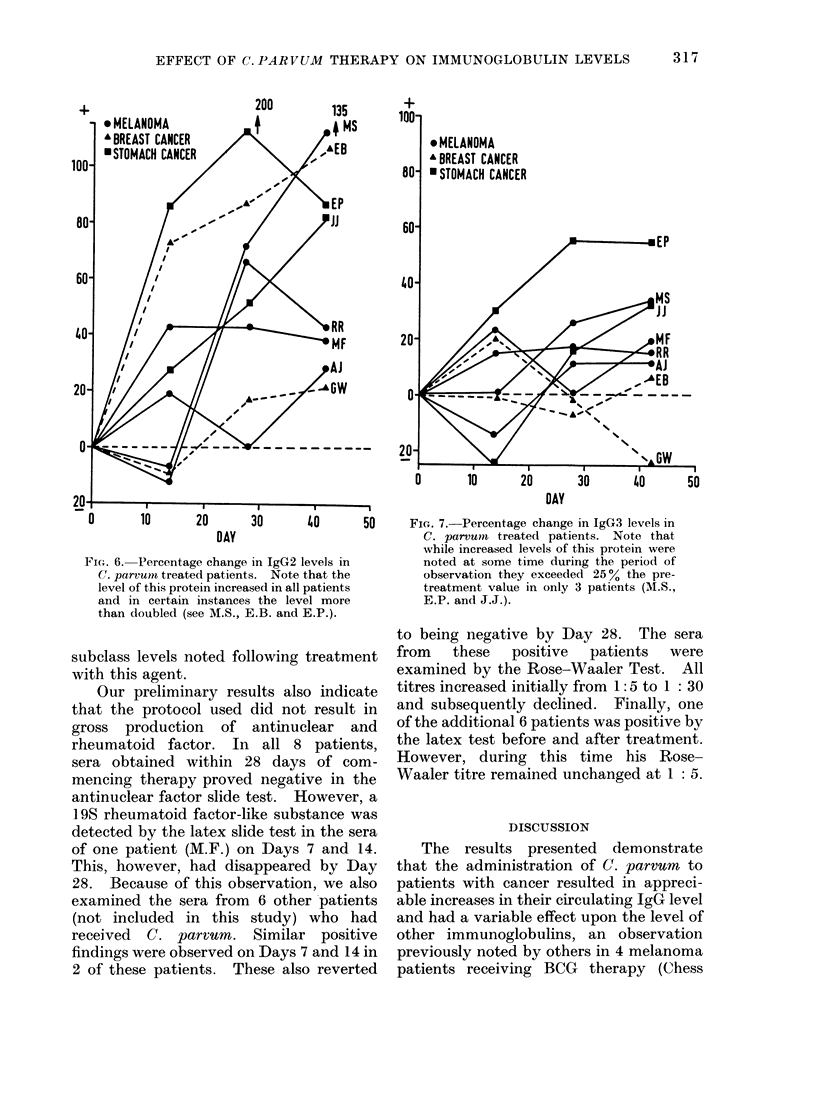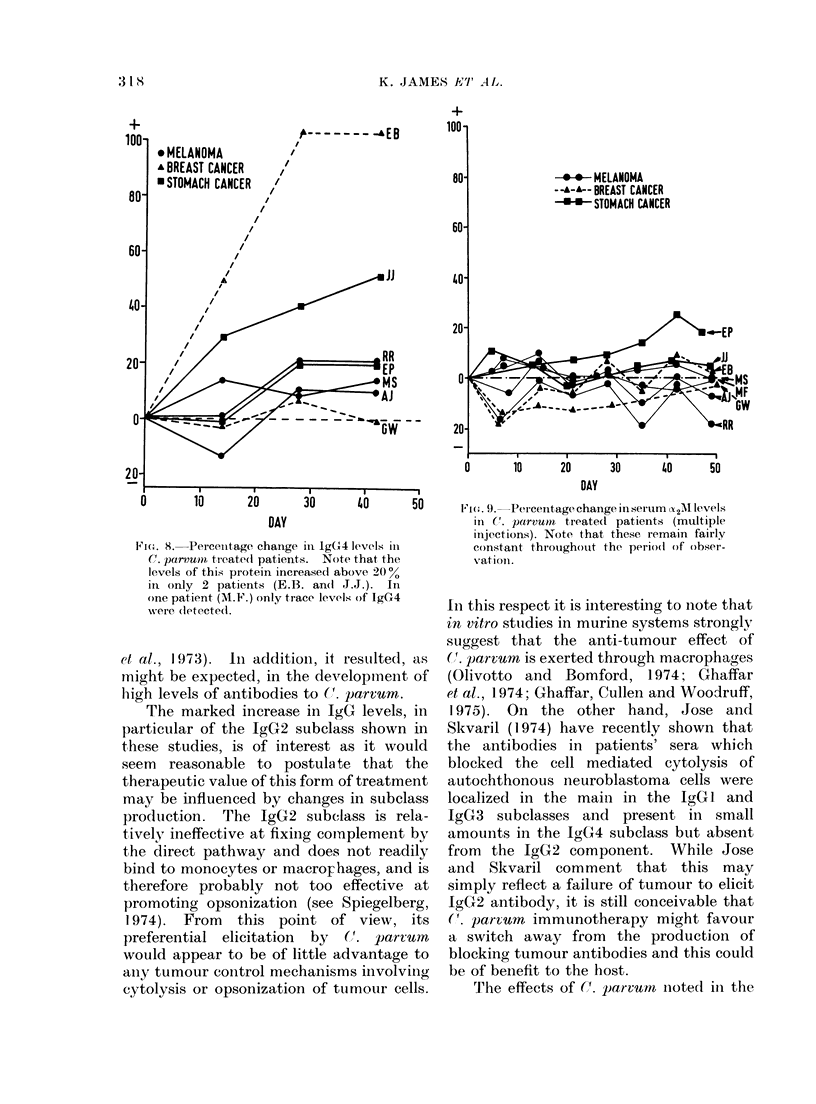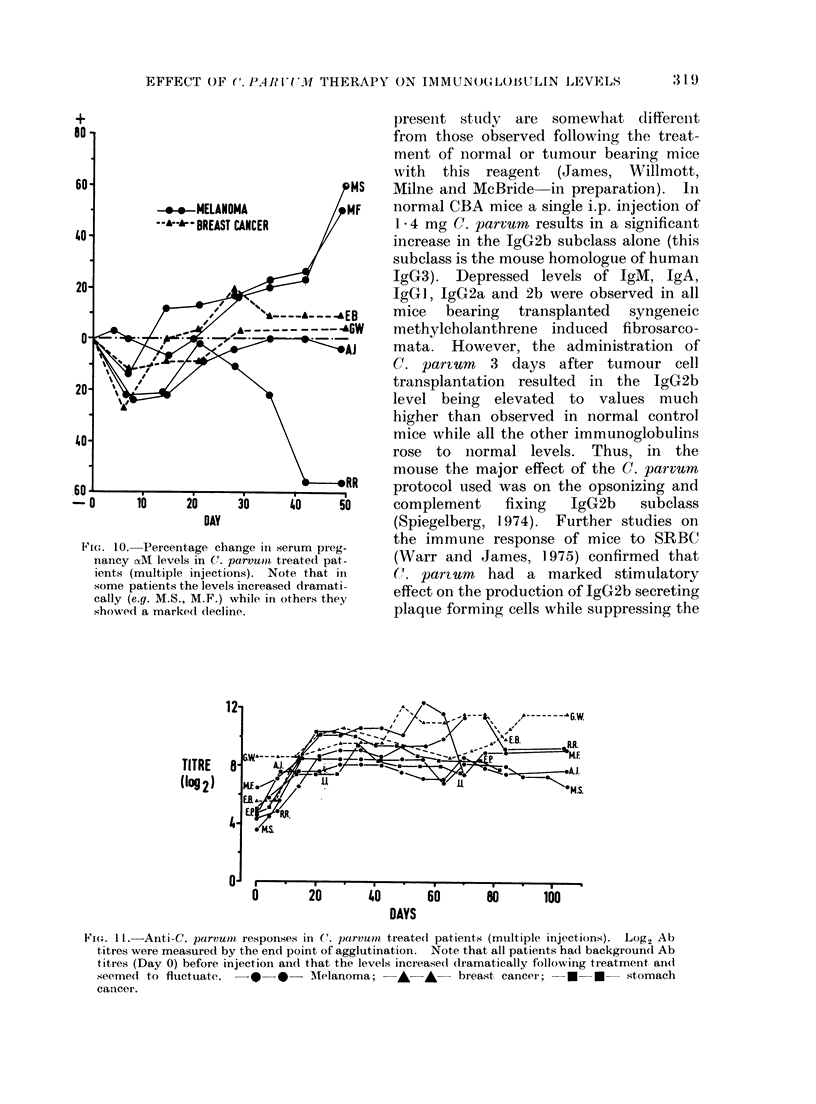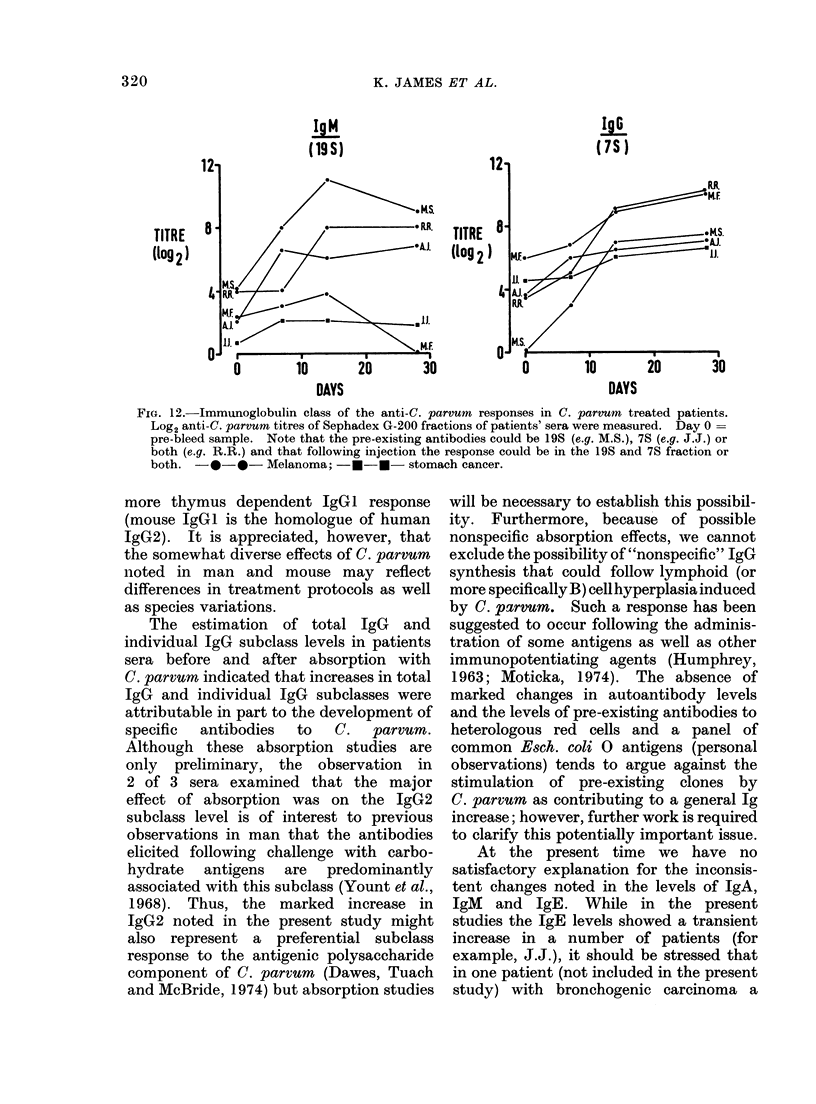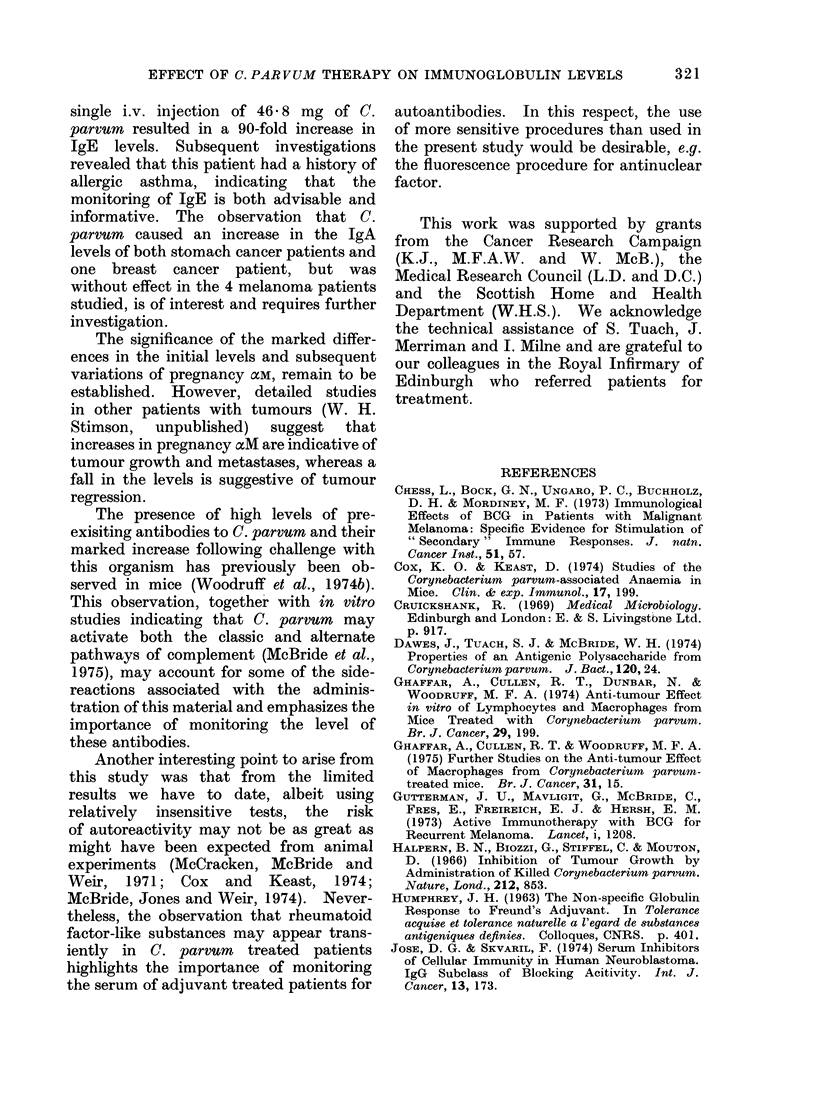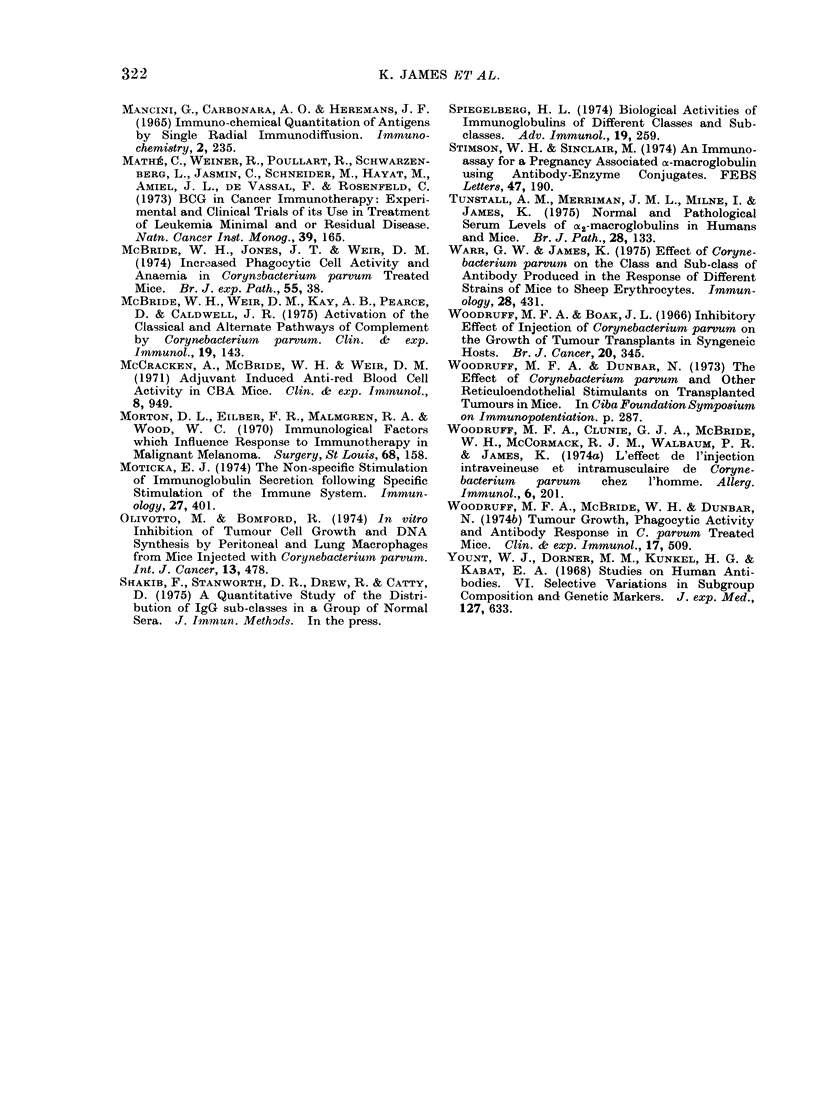# The effect of Corynebacterium parvum therapy on immunoglobulin class and IgG subclass levels in cancer patients.

**DOI:** 10.1038/bjc.1975.229

**Published:** 1975-09

**Authors:** K. James, G. J. Clunie, M. F. Woodruff, W. H. McBride, W. H. Stimson, R. Drew, D. Catty

## Abstract

Detailed serological studies have been undertaken in a small group of cancer patients receiving nonspecific immunotherapy with Corynebacterium parvum (C. parvum). These patients included 4 cases of recurrent malignant melanoma, 2 of stomach cancer and 2 of recurrent breast cancer. They all received an initial i.v. infusion of 20 mg of a formol killed suspension of C. parvum followed by 2 mg (i.m.) at weekly intervals for 10-11 weeks. This protocol consistently resulted in an increase in the circulating IgG levels of all patients but had a variable effect on their IgA, IgM and IgE levels. Increases in the concentration of all 4 IgG subclasses contributed to the overall increase in IgG levels and these changes ranked IgG2 greater than IgG1 greater than IgG3 = IgG4. It also had an inconsistent effect upon the levels of alpha-macroglobulin in pregnancy but the levels of normal serum alpha2-macroglobulin were virtually unchanged. Pre-existing antibodies to C. parvum were noted in all the patients. Titres rose appreciably following C. parvum administration and remained at high, though fluctuating levels, throughout the 100-day period of observation. Absorption studies suggested that the development of antibodies to C. parvum accounted in part for the increased IgG levels noted following this form of therapy. The significance of these changes in relation to the possible anti-tumour effect of C. parvum is discussed.


Br. J. Cancer (1975) **32**, 310

### THE EFFECT OF *CORYNEBACTERIUM PARVUM* THERAPY ON IMMUNOGLOBULIN CLASS AND IgG SUBCLASS LEVELS IN CANCER PATIENTS

K. JAMES,* G. J. A. CLUNIE*, M. F. A. WOODRUFF,* W. H. McBRIDE,* W. H. STIMSON,† R. DREW‡ AND D. CATTY‡

*From the Departments of Surgery and Bacteriology, University of Edinburgh Medical School, Teviot Place, Edinburgh, EH8 9AG*, the Department of Biochemistry, University of Strathclyde, Glasgow† and the Department of Experimental Pathology, University of Birmingham Medical School, Birmingham, B15 2TS‡*

Received 6 May 1975. Accepted 20 May 1975

**Summary.—Detailed serological studies have been undertaken in a small group of cancer patients receiving nonspecific immunotherapy with *Corynebacterium parvum* (*C. parvum*). These patients included 4 cases of recurrent malignant melanoma, 2 of stomach cancer and 2 of recurrent breast cancer. They all received an initial i.v. infusion of 20 mg of a formol killed suspension of *C. parvum* followed by 2 mg (i.m.) at weekly intervals for 10–11 weeks.**

**This protocol consistently resulted in an increase in the circulating IgG levels of all patients but had a variable effect on their IgA, IgM and IgE levels. Increases in the concentration of all 4 IgG subclasses contributed to the overall increase in IgG levels and these changes ranked IgG2 > IgG1 > IgG3 = IgG4. It also had an inconsistent effect upon the levels of α-macroglobulin in pregnancy but the levels of normal serum $\alpha_2$-macroglobulin were virtually unchanged.**

**Pre-existing antibodies to *C. parvum* were noted in all the patients. Titres rose appreciably following *C. parvum* administration and remained at high, though fluctuating levels, throughout the 100-day period of observation. Absorption studies suggested that the development of antibodies to *C. parvum* accounted in part for the increased IgG levels noted following this form of therapy. The significance of these changes in relation to the possible anti-tumour effect of *C. parvum* is discussed.**

DURING recent years, considerable interest has been shown in the use of adjuvants such as BCG for specific and nonspecific immunotherapy of tumours in man (for example, Morton *et al.*, 1970; Mathe *et al.*, 1973; Gutterman *et al.*, 1973). Our own attention has been focussed upon the possible clinical value of formol killed suspensions of *C. parvum*. Detailed studies undertaken in mice have demonstrated convincingly that these preparations can inhibit the growth of transplanted syngeneic tumours of both chemical and viral origin (Woodruff and Boak, 1966; Halpern *et al.*, 1966; Woodruff and Dunbar, 1973). On the basis of the experience gained in animal tumour systems, we have recently administered *C. parvum* to a limited number of cancer patients with poor prognosis. Although it is still too early to assess the clinical value of the treatment used, we report in the present paper the results of detailed serological studies we have undertaken in some of these patients, not only because they are of some fundamental importance but also as a guide to other investigators in this field.

During these studies we have determined the effect of repeated *C. parvum* administration on the levels of immunoglobulin classes and other serum proteins.

Furthermore, because the IgG subclasses (namely IgG1, IgG2, IgG3 and IgG4) differ widely in their *in vitro* and *in vivo* properties (Spiegelberg, 1974), including their capacity to block the cell mediated destruction of tumour cells *in vitro* (Jose and Skvaril, 1974), we have also investigated the effect of *C. parvum* therapy upon individual IgG subclass levels. As far as we are aware, the present results are the first reporting the effects of any form of immunotherapy on subclass levels. Finally, we have also monitored the patients' sera for antibodies to *C. parvum* and have attempted to assess the relevance of these to the immunoglobulin changes.

### MATERIALS AND METHODS

The 8 patients studied comprised 2 with locally recurrent breast cancer following 3–4 years after simple mastectomy and radiotherapy (G.W. and E.B.); 4 with malignant melanoma who, 9 months to 9 years after the initial tumour, had extensive lymph node involvement, local recurrence or multiple subcutaneous metastases, or some combination of these (M.S., M.F., R.B., A.J.); and 2 with carcinoma of the stomach with extensive lymph node involvement who were treated by gastrectomy 9 days (E.P.) and 21 days (J.J.) previously. Clinical data concerning these and other patients treated with *C. parvum* will be reported fully in due course.

The serum protein levels observed before therapy are recorded in Table I. With the exception of G.W. (see later), all patients received an initial i.v. infusion of 20 mg of a formol killed suspension of *C. parvum* followed by 2 mg (i.m.) at weekly intervals for 10–11 weeks. The *C. parvum* used (CN6134 Batch EZ174) was supplied by the Wellcome Research Laboratories, Langley Court, Beckenham. To minimize the febrile reactions which often follow i.v. administration of *C. parvum* (Woodruff *et al.*, 1974*a*), the patients also received aspirin. On one occasion (patient M.S.) the administration of *C. parvum* was temporarily suspended for 2 weeks because of the appearance of a skin rash. One patient (G.W.) had received a single i.v. infusion of 47·6 mg of *C. parvum* some 5 months before commencing the weekly course of intramuscular injections. The serum samples for analysis were always obtained immediately before each *C. parvum* injection and were stored at −20 °C before assay.

The IgM, IgA, IgG, IgG subclass and $\alpha_2$M levels in the sera were determined by the Mancini radial immunodiffusion technique (Mancini, Carbonara and Heremans, 1965). A complete range of standards was included on every plate and all the test samples were assayed in duplicate. The antisera to IgG, IgA and IgM were purchased from Wellcome Reagents Ltd, Langley Court, Beckenham, while the antiserum to $\alpha_2$M was produced in our own laboratory as previously described (Tunstall *et al.*, 1975). The IgG, IgA and IgM reference standards were obtained from Hoechst Pharmaceuticals, Hounslow, while the $\alpha_2$M standard was purchased from Melloy Laboratories, Springfield, Virginia, U.S.A. The preparation and properties of the antisera to the IgG subclasses and the standard antigens used in their assay are fully described elsewhere (Shakib *et al.*, 1975).

The IgE levels were determined by a radioimmunoassay procedure employing the Phadebas IgE test kit (purchased from Pharmacia G.B. Ltd, London). This assay was performed according to the manufacturer's instructions.

The pregnancy $\alpha$-macroglobulin levels were determined by an immunoassay procedure employing antibody–enzyme conjugates. This procedure has been described in detail elsewhere (Stimson and Sinclair, 1974).

Antibodies to *C. parvum* were measured in the sera by a latex agglutination test (Woodruff, McBride and Dunbar, 1974*b*). The results are expressed as $\log_2$ reciprocal of the end point dilution. In certain instances sera were fractionated on Sephadex G-200 columns and the presence of antibody activity in the 19S, 10S, 7S and 4·5S peaks was assessed by the same technique. On other occasions, dilutions of sera (see Tables II and III) were absorbed at 37 °C for 30 min and overnight at 4 °C (×3) with one-tenth of a volume of packed *C. parvum* organisms. Anti-*C. parvum* titres, IgG and IgG subclass levels were measured before and after absorption to give some indication of the amount of IgG which was anti-*C. parvum* antibody.

Screening for antinuclear factors was performed by the qualitative rapid slide test

TABLE II.—*The Effect of Absorption of Patients' Sera with* C. Parvum *Upon IgG Levels*

| Patient | Time of sample | IgG before* absorption† | IgG after absorption | Percent decrease | Pre-trial* IgG |
|---|---|---|---|---|---|
| P.K.‡ | Pre-bleed | 922 | 764 | 17 | — |
| P.K. | Pre-bleed | 1031 | 812 | 19 | — |
| E.P. | Pre-bleed | 1375 | 1161 | 16 | 1027 |
| S.C. | Day 21 | 988 | 549 | 44 | 538 |
| J.J. | Day 27 | 1853 | 1490 | 20 | 1366 |
| R.R. | Day 28 | 1967 | 1509 | 23 | 1434 |
| M.F. | Day 28 | 1595 | 1003 | 37 | 1076 |

* IgG expressed as mg/100 ml.
† Sera were absorbed 3 times at a 1 : 10 dilution with a 1:10 volume of packed organisms. Absorption was carried out at 37 °C for 30 min and overnight at 4 °C.
‡ Patient P.K. was a control.
Note: All anti-*C. parvum* titres after absorption were $<\log_2 2$ AB titre by the agglutination test.

(Hyland, California) while the presence or absence of rheumatoid factor was established by the latex slide test (Hoechst Pharmaceuticals, Hounslow, England). Sera positive in the latex slide test were further examined by the Rose–Waaler procedure employing sensitized sheep cells (Cruickshank, 1969).

### RESULTS

The effect of *C. parvum* therapy on the levels of serum immunoglobulins and other proteins is illustrated in Fig. 1–10. In all the figures the protein levels have been expressed as a percentage of the value observed immediately before therapy (Table I). For ease of presentation, we have plotted only the changes noted during the initial 50 days following commencement of therapy, but in general a similar pattern was apparent over the entire period of observation (70–128 days).

In all cases, the administration of *C. parvum* was accompanied by an increase in the serum IgG level (Fig. 1). This increase was often apparent within 2 weeks of the initial *C. parvum* injection and the levels generally remained elevated throughout the period of treatment. On occasions, the IgG levels increased to almost twice the pretreatment value (see E.B.). The effect of *C. parvum* on the levels of other immunoglobulins was less consistent. While the IgA levels in the melanoma patients were essentially unchanged, marked increases were noted in both stomach cancer patients and one of

FIG. 1.—Percentage change in serum IgG levels in *C. parvum* treated patients (multiple injections). Note that in all patients there was an appreciable increase in IgG levels.

the patients with breast cancer (Fig. 2). On the other hand, elevated IgM levels were observed in 2/4 of the melanoma patients and the 2 stomach cancer patients, but not in the 2 patients with breast cancer. The observations that the increases in IgM levels did not necessarily

FIG. 2.—Percentage change in serum IgA levels in *C. parvum* treated patients (multiple injections). Note that the levels remained fairly constant in melanoma patients but were markedly elevated in both stomach cancer patients and one patient (E.B.) with breast cancer.

FIG. 3.—Percentage change in serum IgM levels in *C. parvum* treated patients (multiple injections). Note the transient and marked changes in some patients, *e.g.* J.J., M.S. and E.P.

occur in the first 2 weeks of therapy and that they were generally transient in nature and could sometimes be followed by a sharp decline (see J.J. and M.S., Fig. 3) were particularly interesting.

The IgE levels showed an initial early increase in 4/7 patients but in all cases this was followed by a decline. Indeed, in 4 cases the IgE levels had fallen well below their pretreatment values within 7 weeks of commencing therapy (see M.F., E.P., E.B. and J.J., Fig. 4). It should be noted that the IgE levels in patient M.S. were less than 4 I.U. in 7/8 assays performed on serum samples obtained within 7 weeks of commencing therapy.

The effect of *C. parvum* therapy upon IgG subclass levels is shown in Fig. 5–8. The IgG1 subclass level increased (29–78%) in all patients except R.R. (Fig. 5) although in 3 patients (M.F., A.J. and G.W.) this was transient. The effect on the IgG2 subclass was more dramatic and lasting (Fig. 6). The levels of this protein were increased in all patients and in 3 individuals more than doubled. Changes in IgG3 and IgG4 levels were less marked, increases greater than 25% being observed in only 3 and 2 patients respectively (Fig. 7, 8).

The *C. parvum* protocol had a variable effect on the levels of the 2 $\alpha$-macroglobulins studied. In general, the serum $\alpha_2$M levels were largely unchanged (Fig. 9), this in itself being a good indication that the changes observed in the levels of other serum proteins were probably not due to haemoconcentration or haemodilution. In contrast, however, variations in pregnancy $\alpha$M levels were noted (Fig. 10). In 5/6 cases there was an initial decline in the level of this protein, followed by a return to pretreatment values in 3 patients, a marked increase in 2 melanoma patients

FIG. 4.—Percentage change in serum IgE levels in *C. parvum* treated patients (multiple injections). Note the initial increase in certain patients (*e.g.* J.J.) followed by a marked decline at later stages (*e.g.* J.J.).

FIG. 5.—Percentage change in IgG1 levels in *C. parvum* treated patients. Note that in all but one patient (R.R.) the IgG1 level increased after the initiation of *C. parvum* therapy. In certain patients this increase was transient (A.J., M.F. and G.W.).

(M.S., M.F.) and a decline in the third patient (R.R.).

All the patients investigated had pre-existing antibodies to *C. parvum* (see Fig. 11). The levels observed were comparable with those noted in a panel of 46 normal donors (23 male and 23 female) aged 51–60 years (mean $\log_2 \pm 1$ s.d. $= 5{\cdot}8 \pm 0{\cdot}9$). The very high levels noted in patient G.W. were undoubtedly attributable to prior treatment with *C. parvum*. Examination of Sephadex G-200 fractions of pretreatment sera indicated that the pre-existing antibodies could be 19S (see M.S., Fig. 12), 7S (see J.J.) or both (see R.R.). Appreciable increases in antibody titre occurred within 2 weeks of commencing therapy, to reach an initial peak titre at 4 weeks (see Fig. 11). Despite repeated i.m. injections, titres tended to fall slightly after the initial peak and in most cases continued to rise and fall in a cyclical manner with a long but uncertain periodicity. The antibodies evoked were again of either the 19S or 7S class (Fig. 12). It should also be noted that the response of certain patients were rather poor (*e.g.*, A.J., J.J. and M.S.).

The absorption of sera with *C. parvum* resulted in an appreciable decrease in the overall IgG levels (Table III) and also affected the levels of individual IgG subclasses (Table III). As was to be expected, this decrease was most apparent in sera obtained following the commencement of therapy. These results suggest that the development of antibodies to *C. parvum* contributes to an appreciable extent to the increased IgG and IgG

FIG. 6.—Percentage change in IgG2 levels in *C. parvum* treated patients. Note that the level of this protein increased in all patients and in certain instances the level more than doubled (see M.S., E.B. and E.P.).

FIG. 7.—Percentage change in IgG3 levels in *C. parvum* treated patients. Note that while increased levels of this protein were noted at some time during the period of observation they exceeded 25% the pre-treatment value in only 3 patients (M.S., E.P. and J.J.).

subclass levels noted following treatment with this agent.

Our preliminary results also indicate that the protocol used did not result in gross production of antinuclear and rheumatoid factor. In all 8 patients, sera obtained within 28 days of commencing therapy proved negative in the antinuclear factor slide test. However, a 19S rheumatoid factor-like substance was detected by the latex slide test in the sera of one patient (M.F.) on Days 7 and 14. This, however, had disappeared by Day 28. Because of this observation, we also examined the sera from 6 other patients (not included in this study) who had received *C. parvum*. Similar positive findings were observed on Days 7 and 14 in 2 of these patients. These also reverted to being negative by Day 28. The sera from these positive patients were examined by the Rose–Waaler Test. All titres increased initially from 1:5 to 1 : 30 and subsequently declined. Finally, one of the additional 6 patients was positive by the latex test before and after treatment. However, during this time his Rose–Waaler titre remained unchanged at 1 : 5.

### DISCUSSION

The results presented demonstrate that the administration of *C. parvum* to patients with cancer resulted in appreciable increases in their circulating IgG level and had a variable effect upon the level of other immunoglobulins, an observation previously noted by others in 4 melanoma patients receiving BCG therapy (Chess

FIG. 8.—Percentage change in IgG4 levels in *C. parvum* treated patients. Note that the levels of this protein increased above 20% in only 2 patients (E.B. and J.J.). In one patient (M.F.) only trace levels of IgG4 were detected.

FIG. 9.—Percentage change in serum $\alpha_2$M levels in *C. parvum* treated patients (multiple injections). Note that these remain fairly constant throughout the period of observation.

*et al.*, 1973). In addition, it resulted, as might be expected, in the development of high levels of antibodies to *C. parvum*.

The marked increase in IgG levels, in particular of the IgG2 subclass shown in these studies, is of interest as it would seem reasonable to postulate that the therapeutic value of this form of treatment may be influenced by changes in subclass production. The IgG2 subclass is relatively ineffective at fixing complement by the direct pathway and does not readily bind to monocytes or macrophages, and is therefore probably not too effective at promoting opsonization (see Spiegelberg, 1974). From this point of view, its preferential elicitation by *C. parvum* would appear to be of little advantage to any tumour control mechanisms involving cytolysis or opsonization of tumour cells. In this respect it is interesting to note that *in vitro* studies in murine systems strongly suggest that the anti-tumour effect of *C. parvum* is exerted through macrophages (Olivotto and Bomford, 1974; Ghaffar *et al.*, 1974; Ghaffar, Cullen and Woodruff, 1975). On the other hand, Jose and Skvaril (1974) have recently shown that the antibodies in patients' sera which blocked the cell mediated cytolysis of autochthonous neuroblastoma cells were localized in the main in the IgG1 and IgG3 subclasses and present in small amounts in the IgG4 subclass but absent from the IgG2 component. While Jose and Skvaril comment that this may simply reflect a failure of tumour to elicit IgG2 antibody, it is still conceivable that *C. parvum* immunotherapy might favour a switch away from the production of blocking tumour antibodies and this could be of benefit to the host.

The effects of *C. parvum* noted in the

Fig. 10.—Percentage change in serum pregnancy αM levels in *C. parvum* treated patients (multiple injections). Note that in some patients the levels increased dramatically (*e.g.* M.S., M.F.) while in others they showed a marked decline.

present study are somewhat different from those observed following the treatment of normal or tumour bearing mice with this reagent (James, Willmott, Milne and McBride—in preparation). In normal CBA mice a single i.p. injection of 1·4 mg *C. parvum* results in a significant increase in the IgG2b subclass alone (this subclass is the mouse homologue of human IgG3). Depressed levels of IgM, IgA, IgG1, IgG2a and 2b were observed in all mice bearing transplanted syngeneic methylcholanthrene induced fibrosarcomata. However, the administration of *C. parvum* 3 days after tumour cell transplantation resulted in the IgG2b level being elevated to values much higher than observed in normal control mice while all the other immunoglobulins rose to normal levels. Thus, in the mouse the major effect of the *C. parvum* protocol used was on the opsonizing and complement fixing IgG2b subclass (Spiegelberg, 1974). Further studies on the immune response of mice to SRBC (Warr and James, 1975) confirmed that *C. parvum* had a marked stimulatory effect on the production of IgG2b secreting plaque forming cells while suppressing the

Fig. 11.—Anti-*C. parvum* responses in *C. parvum* treated patients (multiple injections). $Log_2$ Ab titres were measured by the end point of agglutination. Note that all patients had background Ab titres (Day 0) before injection and that the levels increased dramatically following treatment and seemed to fluctuate. —●—●— Melanoma; —▲—▲— breast cancer; —■—■— stomach cancer.

FIG. 12.—Immunoglobulin class of the anti-*C. parvum* responses in *C. parvum* treated patients. $Log_2$ anti-*C. parvum* titres of Sephadex G-200 fractions of patients' sera were measured. Day 0 = pre-bleed sample. Note that the pre-existing antibodies could be 19S (*e.g.* M.S.), 7S (*e.g.* J.J.) or both (*e.g.* R.R.) and that following injection the response could be in the 19S and 7S fraction or both. —●—●— Melanoma; —■—■— stomach cancer.

more thymus dependent IgG1 response (mouse IgG1 is the homologue of human IgG2). It is appreciated, however, that the somewhat diverse effects of *C. parvum* noted in man and mouse may reflect differences in treatment protocols as well as species variations.

The estimation of total IgG and individual IgG subclass levels in patients sera before and after absorption with *C. parvum* indicated that increases in total IgG and individual IgG subclasses were attributable in part to the development of specific antibodies to *C. parvum*. Although these absorption studies are only preliminary, the observation in 2 of 3 sera examined that the major effect of absorption was on the IgG2 subclass level is of interest to previous observations in man that the antibodies elicited following challenge with carbohydrate antigens are predominantly associated with this subclass (Yount *et al.*, 1968). Thus, the marked increase in IgG2 noted in the present study might also represent a preferential subclass response to the antigenic polysaccharide component of *C. parvum* (Dawes, Tuach and McBride, 1974) but absorption studies will be necessary to establish this possibility. Furthermore, because of possible nonspecific absorption effects, we cannot exclude the possibility of "nonspecific" IgG synthesis that could follow lymphoid (or more specifically B) cell hyperplasia induced by *C. parvum*. Such a response has been suggested to occur following the administration of some antigens as well as other immunopotentiating agents (Humphrey, 1963; Moticka, 1974). The absence of marked changes in autoantibody levels and the levels of pre-existing antibodies to heterologous red cells and a panel of common *Esch. coli* O antigens (personal observations) tends to argue against the stimulation of pre-existing clones by *C. parvum* as contributing to a general Ig increase; however, further work is required to clarify this potentially important issue.

At the present time we have no satisfactory explanation for the inconsistent changes noted in the levels of IgA, IgM and IgE. While in the present studies the IgE levels showed a transient increase in a number of patients (for example, J.J.), it should be stressed that in one patient (not included in the present study) with bronchogenic carcinoma a single i.v. injection of 46·8 mg of *C. parvum* resulted in a 90-fold increase in IgE levels. Subsequent investigations revealed that this patient had a history of allergic asthma, indicating that the monitoring of IgE is both advisable and informative. The observation that *C. parvum* caused an increase in the IgA levels of both stomach cancer patients and one breast cancer patient, but was without effect in the 4 melanoma patients studied, is of interest and requires further investigation.

The significance of the marked differences in the initial levels and subsequent variations of pregnancy αM, remain to be established. However, detailed studies in other patients with tumours (W. H. Stimson, unpublished) suggest that increases in pregnancy αM are indicative of tumour growth and metastases, whereas a fall in the levels is suggestive of tumour regression.

The presence of high levels of pre-exisiting antibodies to *C. parvum* and their marked increase following challenge with this organism has previously been observed in mice (Woodruff *et al.*, 1974*b*). This observation, together with *in vitro* studies indicating that *C. parvum* may activate both the classic and alternate pathways of complement (McBride *et al.*, 1975), may account for some of the side-reactions associated with the administration of this material and emphasizes the importance of monitoring the level of these antibodies.

Another interesting point to arise from this study was that from the limited results we have to date, albeit using relatively insensitive tests, the risk of autoreactivity may not be as great as might have been expected from animal experiments (McCracken, McBride and Weir, 1971; Cox and Keast, 1974; McBride, Jones and Weir, 1974). Nevertheless, the observation that rheumatoid factor-like substances may appear transiently in *C. parvum* treated patients highlights the importance of monitoring the serum of adjuvant treated patients for autoantibodies. In this respect, the use of more sensitive procedures than used in the present study would be desirable, *e.g.* the fluorescence procedure for antinuclear factor.

This work was supported by grants from the Cancer Research Campaign (K.J., M.F.A.W. and W. McB.), the Medical Research Council (L.D. and D.C.) and the Scottish Home and Health Department (W.H.S.). We acknowledge the technical assistance of S. Tuach, J. Merriman and I. Milne and are grateful to our colleagues in the Royal Infirmary of Edinburgh who referred patients for treatment.

### REFERENCES

CHESS, L., BOCK, G. N., UNGARO, P. C., BUCHHOLZ, D. H. & MORDINEY, M. F. (1973) Immunological Effects of BCG in Patients with Malignant Melanoma: Specific Evidence for Stimulation of "Secondary" Immune Responses. *J. natn. Cancer Inst.*, **51**, 57.

COX, K. O. & KEAST, D. (1974) Studies of the *Corynebacterium parvum*-associated Anaemia in Mice. *Clin. & exp. Immunol.*, **17**, 199.

CRUICKSHANK, R. (1969) *Medical Microbiology.* Edinburgh and London: E. & S. Livingstone Ltd. p. 917.

DAWES, J., TUACH, S. J. & MCBRIDE, W. H. (1974) Properties of an Antigenic Polysaccharide from *Corynebacterium parvum. J. Bact.*, **120**, 24.

GHAFFAR, A., CULLEN, R. T., DUNBAR, N. & WOODRUFF, M. F. A. (1974) Anti-tumour Effect *in vitro* of Lymphocytes and Macrophages from Mice Treated with *Corynebacterium parvum. Br. J. Cancer*, **29**, 199.

GHAFFAR, A., CULLEN, R. T. & WOODRUFF, M. F. A. (1975) Further Studies on the Anti-tumour Effect of Macrophages from *Corynebacterium parvum*-treated mice. *Br. J. Cancer*, **31**, 15.

GUTTERMAN, J. U., MAVLIGIT, G., MCBRIDE, C., FRES, E., FREIREICH, E. J. & HERSH, E. M. (1973) Active Immunotherapy with BCG for Recurrent Melanoma. *Lancet*, i, 1208.

HALPERN, B. N., BIOZZI, G., STIFFEL, C. & MOUTON, D. (1966) Inhibition of Tumour Growth by Administration of Killed *Corynebacterium parvum. Nature, Lond.*, **212**, 853.

HUMPHREY, J. H. (1963) The Non-specific Globulin Response to Freund's Adjuvant. In *Tolerance acquise et tolerance naturelle a l'egard de substances antigeniques definies.* Colloques, CNRS. p. 401.

JOSE, D. G. & SKVARIL, F. (1974) Serum Inhibitors of Cellular Immunity in Human Neuroblastoma. IgG Subclass of Blocking Acitivity. *Int. J. Cancer*, **13**, 173.

MANCINI, G., CARBONARA, A. O. & HEREMANS, J. F. (1965) Immuno-chemical Quantitation of Antigens by Single Radial Immunodiffusion. *Immunochemistry*, **2**, 235.

MATHÉ, C., WEINER, R., POULLART, R., SCHWARZENBERG, L., JASMIN, C., SCHNEIDER, M., HAYAT, M., AMIEL, J. L., DE VASSAL, F. & ROSENFELD, C. (1973) BCG in Cancer Immunotherapy: Experimental and Clinical Trials of its Use in Treatment of Leukemia Minimal and or Residual Disease. *Natn. Cancer Inst. Monog.*, **39**, 165.

MCBRIDE, W. H., JONES, J. T. & WEIR, D. M. (1974) Increased Phagocytic Cell Activity and Anaemia in *Corynebacterium parvum* Treated Mice. *Br. J. exp. Path.*, **55**, 38.

MCBRIDE, W. H., WEIR, D. M., KAY, A. B., PEARCE, D. & CALDWELL, J. R. (1975) Activation of the Classical and Alternate Pathways of Complement by *Corynebacterium parvum*. *Clin. & exp. Immunol.*, **19**, 143.

MCCRACKEN, A., MCBRIDE, W. H. & WEIR, D. M. (1971) Adjuvant Induced Anti-red Blood Cell Activity in CBA Mice. *Clin. & exp. Immunol.*, **8**, 949.

MORTON, D. L., EILBER, F. R., MALMGREN, R. A. & WOOD, W. C. (1970) Immunological Factors which Influence Response to Immunotherapy in Malignant Melanoma. *Surgery, St Louis*, **68**, 158.

MOTICKA, E. J. (1974) The Non-specific Stimulation of Immunoglobulin Secretion following Specific Stimulation of the Immune System. *Immunology*, **27**, 401.

OLIVOTTO, M. & BOMFORD, R. (1974) *In vitro* Inhibition of Tumour Cell Growth and DNA Synthesis by Peritoneal and Lung Macrophages from Mice Injected with *Corynebacterium parvum*. *Int. J. Cancer*, **13**, 478.

SHAKIB, F., STANWORTH, D. R., DREW, R. & CATTY, D. (1975) A Quantitative Study of the Distribution of IgG sub-classes in a Group of Normal Sera. *J. Immun. Methods*. In the press.

SPIEGELBERG, H. L. (1974) Biological Activities of Immunoglobulins of Different Classes and Subclasses. *Adv. Immunol.*, **19**, 259.

STIMSON, W. H. & SINCLAIR, M. (1974) An Immunoassay for a Pregnancy Associated $\alpha$-macroglobulin using Antibody-Enzyme Conjugates. *FEBS Letters*, **47**, 190.

TUNSTALL, A. M., MERRIMAN, J. M. L., MILNE, I. & JAMES, K. (1975) Normal and Pathological Serum Levels of $\alpha_2$-macroglobulins in Humans and Mice. *Br. J. Path.*, **28**, 133.

WARR, G. W. & JAMES, K. (1975) Effect of *Corynebacterium parvum* on the Class and Sub-class of Antibody Produced in the Response of Different Strains of Mice to Sheep Erythrocytes. *Immunology*, **28**, 431.

WOODRUFF, M. F. A. & BOAK, J. L. (1966) Inhibitory Effect of Injection of *Corynebacterium parvum* on the Growth of Tumour Transplants in Syngeneic Hosts. *Br. J. Cancer*, **20**, 345.

WOODRUFF, M. F. A. & DUNBAR, N. (1973) The Effect of *Corynebacterium parvum* and Other Reticuloendothelial Stimulants on Transplanted Tumours in Mice. In *Ciba Foundation Symposium on Immunopotentiation*. p. 287.

WOODRUFF, M. F. A., CLUNIE, G. J. A., MCBRIDE, W. H., MCCORMACK, R. J. M., WALBAUM, P. R. & JAMES, K. (1974*a*) L'effect de l'injection intraveineuse et intramusculaire de *Corynebacterium parvum* chez l'homme. *Allerg. Immunol.*, **6**, 201.

WOODRUFF, M. F. A., MCBRIDE, W. H. & DUNBAR, N. (1974*b*) Tumour Growth, Phagocytic Activity and Antibody Response in *C. parvum* Treated Mice. *Clin. & exp. Immunol.*, **17**, 509.

YOUNT, W. J., DORNER, M. M., KUNKEL, H. G. & KABAT, E. A. (1968) Studies on Human Antibodies. VI. Selective Variations in Subgroup Composition and Genetic Markers. *J. exp. Med.*, **127**, 633.